# Chronic periodontitis in a patient with multiple sclerosis—A case report

**DOI:** 10.1002/ccr3.9261

**Published:** 2024-08-06

**Authors:** Rayeheh Tavajohi, Amitis Sarbaz, Hooshyar Honarmand, Abdorreza Naser Moghadasi

**Affiliations:** ^1^ Department of Clinical Pharmacy, School of Pharmacy Tehran University of Medical Sciences Tehran Iran; ^2^ Georgia School of Orthodontics Atlanta Georgia USA; ^3^ Multiple Sclerosis Research Center Neuroscience Institute Tehran University of Medical Sciences Tehran Iran

**Keywords:** case report, chronic periodontitis, multiple sclerosis, teeth loss

## Abstract

Multiple sclerosis (MS) is one of the common central nervous system diseases, but it can cause dysfunction in other organs such as periodontal tissues. However, it has not been as noticeable. This report aimed to present a 44‐year‐old patient with severe chronic periodontitis, and sudden teeth loss since the diagnosis of MS.

## 1 INTRODUCTION

Although the exact cause of multiple sclerosis (MS), a chronic inflammatory disease of the central nervous system, is unclear, genetic and environmental variables, including smoking, blood vitamin D levels, and Epstein–Barr virus (EBV) infection, may have a significant impact on the illness' pathophysiology.^1^ EBV which belongs to the herpes family affects more than 90% of worldwide population, and it lives in the body during the life after primary infection. In addition to its role in MS pathogenesis, it can cause periodontitis.^2^ Periodontitis is an inflammatory and infectious condition that affects teeth‐supporting tissues, which is the main reason for teeth loss in adulthood.^3^

The relationship between MS and periodontitis is not fully understood. Periodontitis may be related to MS pathogenesis and exacerbation.^4^ Moreover, medications can cause periodontitis as well.^5^ This article aimed to report a case of relapsing–remitting MS (RRMS) with the complaint of sudden teeth loss twice since the diagnosis of MS.

## 2 CASE PRESENTATION

A 44‐year‐old female with RRMS presented at MS clinic for a routine follow‐up, complaining of tooth loss after the last ocrelizumab administration.

Her first symptoms of paresthesia, blurred vision and gum pain started in 2013 after a stressful personal situation. She visited the dentist complaining gum pain but no abnormalities were detected. She had no other underlying diseases except for postpartum depression and was not taking any medication. Family and habitual histories were negative. RRMS was first diagnosed in February 2016 with symptoms of complete hemiparesis and Lhermitte's sign. She stated that one of her teeth fell out during sleep after this attack. After being admitted to the hospital, the patient received pulse doses of methylprednisolone. She had been started on three times a week of interferon β‐1a as a disease‐modifying medication. Interferon β‐1a was discontinued in November 2016, in terms of severe depression, and glatiramer acetate was initiated, which led to improvement in psychological condition. In October 2021, she had an MS attack with complete paraparesis as a result of medication non‐compliance and discontinuation of glatiramer acetate for 1 year. After hospital admission and administration of methylprednisolone for the management of MS exacerbation, ocrelizumab was initiated for her in May 2022. Four days after the second administration of ocrelizumab in November 2022, another tooth fell out. Table 1 summarizes her clinical events.

**TABLE 1:** Outline of clinical events.

December 2013	First symptoms of paresthesia, gum pain, blurred vision
February 2016	Lhermitte sign and hemiparesis, sudden tooth loss, pulse doses of methylprednisolon administration, initiation of interferon β‐1a
November 2016	Severe depression, switch from interferon β‐1a to glatiramer acetate
November 2020	Discontinuotion of glatiramer acetate by the patient herself in terms of non‐compliance
October 2021	MS exacerbation with the manifestation of paraparesis, hospital admission, pulse doses of methylprednisolon administration
May 2022	Ocrelizumab initiation
November 2022	Sudden tooth fall after the second dose of ocrelizumab
August 2023	Dentistry visit, OPG performed

Abbreviations: MS, multiple sclerosis; OPG, orthopantomogram.

She stated that her oral hygiene was good. No gingival bleeding occurred for her. Family history of periodontitis was negative. Besides her disease‐modifying medication, she also had taken vitamin D and sertraline. The patient was fully ambulatory, and her calculated expanded disability status scale (EDSS) was 2.5.

## 3 METHODS

She visited the dentist in August 2023, and a panoramic image was requested to perform.

**FIGURE 1:**
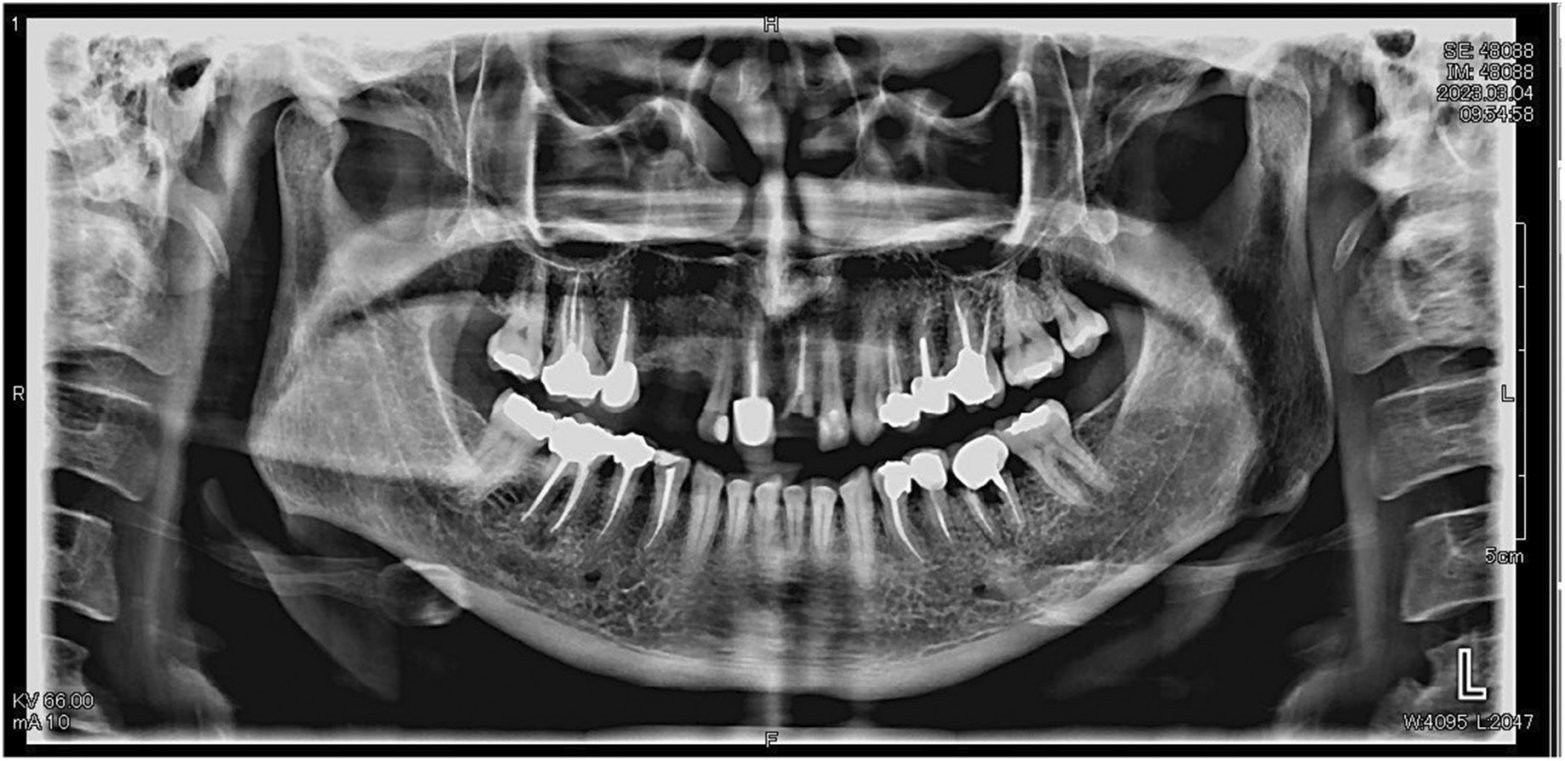
Horizontal bone loss in the upper and lower anterior and posterior region. Thinner condyle on the left side. Missing teeth on 1.3, 1.4, 1.8, 3.8, and 4.8 area.

Based on her panoramic image taken on August 4, 2023, the patient had severe horizontal bone loss in the anterior section of the upper and lower arch, as well as missing teeth 1.3, 1.4, 1.8, 3.8, and 4.8. She had root canals done on tooth 1.6, 1.5, 1.1, 2.1, 2.4, 2.5, and 2.6 along with crowns, as well as 3.4, 3.5, 3.6, 4.4, 4.5, and 4.6. She had restorations on teeth 1.7, 2.7, 3.7, and 4.7. Her left condyle was thinner than her right condyle in the following panoramic image (Figure 1).

**FIGURE 2:**
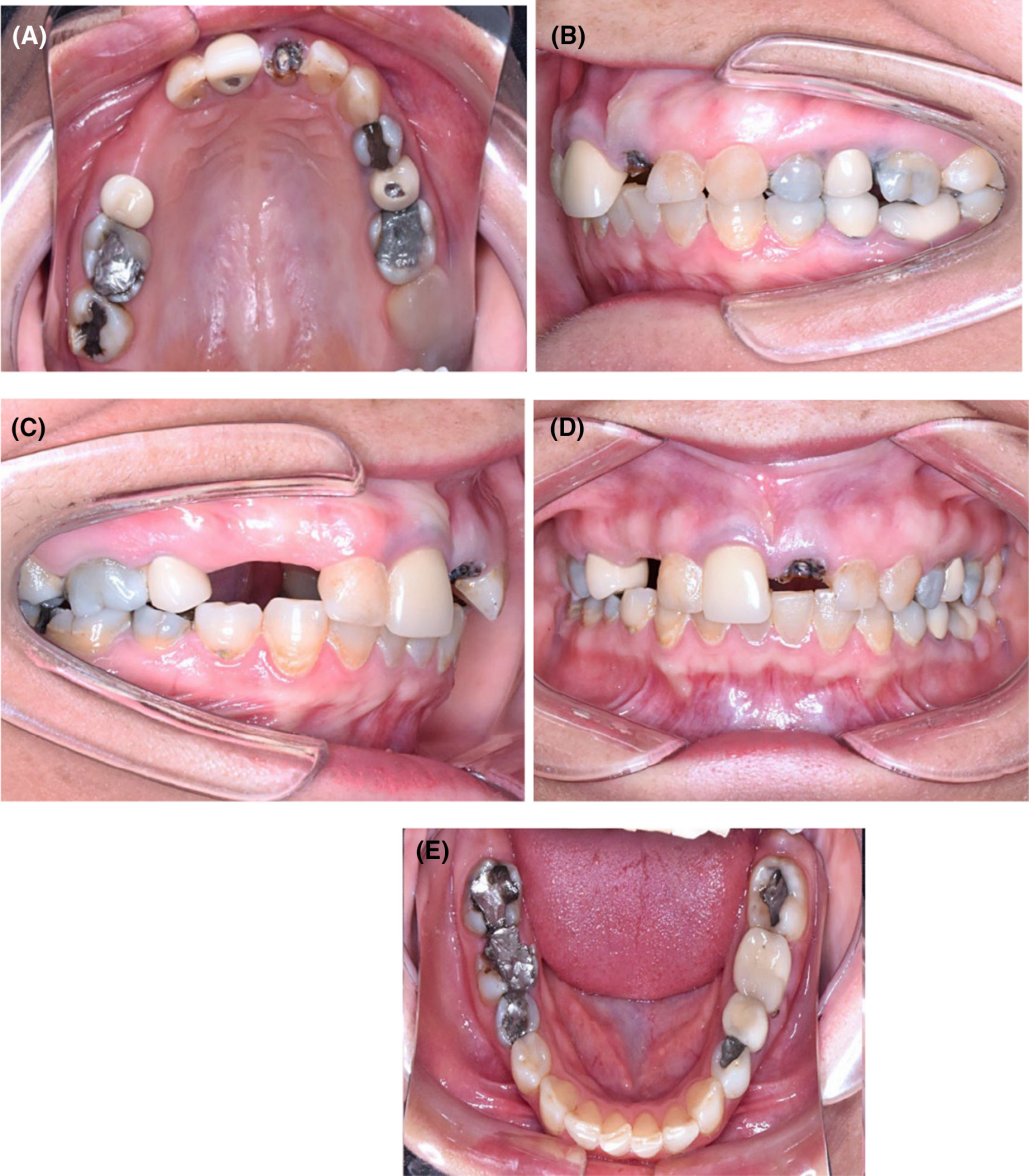
Intraoral images (A–E): (A) maxillary arch illustrating missing teeth and several teeth with multiple restorations; (B–E) illustrate pigmentations due to amalgam restorations, discolored teeth, missing and carious teeth, as well as recessions on the lower canines.

The discoloration and amalgam pigmentation were very prominent in her intraoral photos (Figure 2). Based on her periodontal examination, periodontal recession was majorly around 3.3 and 3.4, and in other areas. The average of probing depth, bleeding on probing index, plaque index, and gingival index was 4 mm, 4.5%, 7%, and 0.8, respectively. The decayed, missed, filled tooth (DMFT) was 24 (D: 4, M: 2, and F:18, excluding wisdom teeth). Her periodontal hygiene was good.

## 4 RESULTS AND CONCLUSION

She was diagnosed with severe chronic periodontitis. The main treatment is scaling and root planning on a routine basis and followed up for every 3 months. The patient was not compliant with her follow‐ups; however, she did visit for multiple loosened teeth.

In order to determine if MS and periodontal disease are related, individuals with MS should be evaluated from the perspective of periodontitis both before and during therapy with disease‐modifying medications as well as during exacerbations of MS. Additionally, MS may present as periodontal disease initially.

## 5 DISCUSSION

The purpose of this case study was to report a 44‐year‐old female with RRMS who developed chronic periodontitis and sudden teeth loss since the diagnosis of MS.

MS patients may experience orofacial manifestations, including vision problems, temporomandibular joint disorder, dysarthria, dysphagia, facial palsy, trigeminal neuralgia, and oral and perioral paresthesia.^1^ Gingival pain was the initial manifestation of MS in our case report. She encountered abrupt tooth loss subsequent to her initial exacerbation of MS, devoid of any prior records of hemorrhage or trauma. An additional loss of teeth transpired 6 years subsequent to the administration of the second dose of ocrelizumab.

One of the interesting issues, nowadays, is the association between neurodegenerative disease and oral microbiome. So, any dysfunction in the oral microbiota‐brain axis can cause MS, Parkinson disease, and Alzheimer disease.^6^ Therefore, screening and prevention of neurodegenerative disease may begin with dentistry. Dysbiosis of oral bacteria can generate systemic inflammation in terms of the release of proinflammatory cytokines. These cytokines can pass the blood–brain barrier via the trigeminal nerve into the brain. According to Buccellato's study, the oral microbiome changes in MS, and wearable mouthguard biosensors can predict MS attacks.^7^

Chronic periodontitis (CP) that is diagnosed for our patient is studied in MS disease. The parameters that are in agreement with the present diagnosis in our case were probing depth (PD) = 4 mm, bleeding on probing (BOP) index <10%, and the presence of radiographic bone loss without a history of periodontal treatment.^8^ CP is a chronic inflammatory disease in dental supportive structures with microbial etiology like EBV.^4^ EBV infection can increase the risk of MS disease 32 times more than healthy people.^9^ Therefore, the etiology of CP may be linked to the onset or aggravation of MS. Case–control research conducted in Taiwan found that only female patients had a correlation between MS and periodontitis.^4^ A systematic review performed in 2023, concluded that CP is more prevalent in MS patients compared to healthy people. Sphingosine‐1‐phosphate can increase the risk of CP so fingolimod may be the drug of choice for MS patients with CP.^10^ On‐time referral to a dentist is crucial to prevent further teeth loss in CP.^11^ Unfortunately, our patient did not visit the dentist after the first tooth loss. Moreover, her compliance with frequent visits after the CP diagnosis was not good. So, this can lead to an incomplete treatment process of CP and probable further teeth loss.

Inadequate oral hygiene, fewer dental referrals, and adverse drug reactions are other important parameters that encountered MS patients with an increased risk of developing dental and gingival disorders.^5^ Weariness and trouble walking might make it difficult to do regular tasks like brushing and flossing their teeth and scheduling regular dental checkups.^5^ There is an association between the degree of physical disability that is measured by EDSS and dental‐periodontal conditions. Plaque index, probing depth, and gingival index occur with a higher incidence in the patients with high physical disability.^12^ However, MS patients with low EDSS scores and no difficulties to perform oral hygiene had higher bleeding on probing index, mean periodontal pocket depth, and higher expression of interleukin 2 in biofilm samples compared to healthy control group. So, inflammation itself plays a crucial role in creating the periodontal problems in MS patients.^13^ In this scenario, our patient had a low EDSS score, and her oral hygiene was good, but she had experienced dental‐periodontal problems since the initiation of her MS diagnosis.

Medications that MS patients were taken during their life affect the oral health status. Symptom management therapy with TCA, and other drugs like tolterodine, pregabalin, and dalfampridine can cause xerostomia, and have negative effects on dental health.^14^ The impact of immunomodulators on oral health remains controversial. In 2018, Vacchiano et al., reported three cases of MS patients who experienced teeth loss during the treatment with teriflunomide. Teriflunomide was discontinued in these patients and cholestyramine was administered in two cases.^15^ Another case study reported a case of necrotizing periodontitis with severe gingival pain during the treatment with ocrelizumab.^16^ It is concluded from both studies that orodental status should be assessed before and during the treatment with these immunosuppressants.^15^, ^16^ A case of MS patient with gingival inflammation and bleeding was reported in 2023. The disease modifying agent that was used for this patient was ocrelizumab. After the diagnosis of gingival inflammation, amoxicillin–clavulonate 625 mg twice daily plus metronidazole 500 mg twice daily were prescribed. Chlorhexidine mouthwash was used and consequently, great improvement was seen in the gingival condition.^14^ Long‐term use of anti‐CD20 agents, such as rituximab and ocrelizumab, can cause gingival recession, gingivitis, and periodontitis as a result of B‐cell depletion, IgA deficiency, and the induction of oral dysbiosis.^17^ Contrary to previous studies, Felhofer et al. declared that glatiramer acetate, mitoxantrone, natalizumab, and ocrelizumab show some positive effects through the downregulation of matrix metalloproteinase (MMPs). The overexpression of MMP 7 and 9 can be seen in both periodontitis and MS. MMP 9 was significantly lower in the subgingival plaque samples of the MS group due to the suppressive effects of disease‐modifying medications on the expression of MMPs.^13^ In this scenario, no subgingival plaque samples were collected to assess the expression of inflammatory parameters. Besides, the patient of this case study experienced the first tooth loss when she did not get any disease modifying agents. The second tooth loss happened during the treatment with ocrelizumab. So, it is not clear whether ignoring the dental‐periodontal status had led to further tooth loss or ocrelizumab had exacerbated the periodontitis.

Our patient experienced depression that can affect oral health. Nangle et al. concluded that oral health issues in MS are related to mental health. Depression and anxiety can have negative effects on oral health.^18^ A higher risk of periodontitis can be seen in these psychological diseases.^19^

Sufficient levels of vitamin D supports periodontal health so a low level of vitamin D can increase the severity of periodontitis.^20^ However, our patient had a sufficient level of vitamin D during follow‐ups.

## AUTHOR CONTRIBUTIONS

**Rayeheh Tavajohi:** Data curation; writing – original draft. **Amitis Sarbaz:** Investigation; writing – original draft. **Hooshyar Honarmand:** Conceptualization; supervision. **Abdorreza Naser Moghadasi:** Conceptualization; supervision; writing – review and editing.

## FUNDING INFORMATION

This study was not supported by any funding.

## CONFLICT OF INTEREST STATEMENT

The authors declare that they have no conflict of interest.

## CONSENT

Written informed consent has been obtained from the patient to publish this report in accordance with the journal's patient consent policy.

## Data Availability

The data are available from the corresponding author upon reasonable request.
